# Cost-Effectiveness of a Solo Anesthesiologist During Transcatheter Aortic Valve Replacement in Patients With Severe Comorbidities

**DOI:** 10.7759/cureus.90398

**Published:** 2025-08-18

**Authors:** Kimberly L Skidmore, Alan D Kaye, Kamian M Buggage, Carliss M Sampognaro, Ellie MacDonald, Rachael Bartolina, Sydney Mashaw, Giustino Varrassi, Sahar Shekoohi, Shahab Ahmadzadeh

**Affiliations:** 1 Department of Anesthesiology, School of Medicine, Louisiana State University Health Sciences Center, Shreveport, USA; 2 Department of Pain Medicine, Fondazione Paolo Procacci, Rome, ITA

**Keywords:** aortic stenosis, congestive heart failure, conscious sedation, general anesthesia, monitored anesthesia care, multidisciplinary preoperative evaluation, obstructive sleep apnea, pulmonary hypertension, staffing cardiac anesthesiologists, transcatheter aortic valve replacement

## Abstract

The present investigation evaluates risk factors and the timing of conversion to general anesthesia (GA) instead of conscious sedation (CS) for patients undergoing transcatheter aortic valve replacement (TAVR). The femoral artery approach to TAVR is primarily used for older patients with aortic stenosis. CS without an anesthesiologist involved in many situations is becoming the standard of care due to faster recovery, fewer complications, and lower costs, concurrent with easier placement of smaller new-generation valves. GA remains necessary for patients with specific health conditions that may complicate spontaneous breathing or trigger cardiovascular collapse. Emergent conversion to GA carries a 30% mortality rate. Consequently, monthly multidisciplinary planning meetings should include a cardiac anesthesiologist to identify risk factors that necessitate monitored anesthesia care (MAC) administered by the same solo physician, rather than CS administered by registered nurses under the direction of a cardiologist. These insights will help guide future pathways for triage of obstructive sleep apnea (OSA) and congestive heart failure (CHF), especially pulmonary hypertension, toward earlier therapies of pressure support noninvasive ventilation, gradual vasopressors, and sedatives titrated in addition to predominantly dexmedetomidine.

## Introduction and background

Aortic stenosis (AS) treated with transcatheter aortic valve replacement (TAVR), initially developed in 2002, required general anesthesia (GA). By 2016, conscious sedation (CS) had become the preferred method [1]. A recent meta-analysis demonstrated that GA was associated with a higher use of vasopressors and longer hospital length of stay compared to the two alternative anesthetics. One alternative to GA is CS administered by a registered nurse (RN). Another alternative is monitored anesthesia care (MAC), which is defined as an anesthesiologist administering medications [2]. Following a standardized manpower and anesthesia technique algorithm can have a significant financial impact.

Previous studies were biased by the chicken-or-egg phenomenon, which neither retrospective nor prospective trials could compensate for with propensity matching. While techniques improved, allowing most clinicians to choose CS, critically ill patients still required GA. In this regard, conversion rates from CS to GA remain approximately 4%, with little detail available about long-term outcomes or the urgency with which hypotension necessitated some conversions to GA. We aimed, therefore, to describe the risk criteria for degrees of congestive heart failure (particularly pulmonary hypertension) that should trigger MAC by a solo cardiac anesthesiologist rather than CS. We conclude this narrative review by examining the cost-effectiveness of working solo when fewer errors occur during handoffs.

CS is contraindicated if continuous transesophageal echocardiography (TEE) is required to improve quality compared to transthoracic echocardiography (TTE). Additionally, CS is not ideal when approaching cannulation via the subclavian artery or transapical route, rather than via the femoral artery [1,3]. A laryngeal mask airway (LMA) is a middle-of-the-road option for GA, without incurring significantly more risk than CS, utilized if a patient shows some obstruction to the airway during MAC. We also aimed to describe when to invoke less or more invasive options than LMA for patients with obstructive sleep apnea (OSA). TAVR is performed in a hybrid operating room, equipped with both catheterization laboratory and surgical capabilities, where GA becomes necessary before instituting cardiopulmonary bypass or extensive femoral artery surgery [1,3]. Preoperative planning meetings should consider these risk factors to avoid emergency conversion to GA.

Historically, TAVR was performed under GA related to the needs of TEE to guide the procedure [4-6]. No longer is this believed necessary. Paravalvular leak (PVL), the most common long-term adverse outcome, is more accurately diagnosed immediately after the procedure with TEE than with TTE [7,8]. Patients receiving a postoperative day one TTE were compared to another group who had only one intraoperative TTE. There was no difference in rates of tamponade, mortality, or urgent TEE rates (with an incidence of approximately 4%) or 30-day mortality (approximately 1%) when the postoperative day-one TTE was skipped [9]. At a one-year follow-up, in over 450 consecutive patients in 2014, the intraoperative TTE was as effective as the postoperative day one TTE in detecting PVL, paving the way for same-day discharge to home. Before 2017, all 1,200 TAVR procedures at one center had both CS and TTE. Among those converting to GA, 1.9% did so because of better views generally with TEE, and 25% to evaluate PVL with TEE; however, the majority switched to GA due to severe hemodynamic compromise [8]. The readmission rate within 30 days was also similar, at 8%. No statistically significant change in PVL or patient-prosthesis mismatch was found; however, trends may have emerged in this potentially underpowered study, which compared TEE with TTE. Their population appeared healthier than recently encountered, with only 1% on dialysis and 4% having an ejection fraction (EF) of less than 30%.

## Review

Risk stratification for TAVR anesthesia techniques

Obstructive Sleep Apnea and Morbid Obesity

There have been many parallel lessons learned in endoscopy with obstructive sleep apnea (OSA). OSA is more closely linked to airway obstruction than body mass index (BMI) [10]. Lessons learned from sedating obese patients for gastric endoscopy mimic portions of the stress of TAVR and are identical to stress during insertion of a TEE probe. Specifically, hypoxemia was reduced in a prospective, randomized trial of bilevel positive airway pressure (BiPAP) (a device for pressure support ventilation) compared to nasal prongs in individuals with a BMI over 25 or a STOP-BANG (OSA survey score) of over 310. Another study demonstrated that pulmonary disease contributes to a 2.5-fold higher risk of sedation-related adverse events (SRAEs) [11]. These investigators also discovered that diabetes mellitus, infections, dialysis, and especially (two-fold risk) coronary artery disease (CAD) or congestive heart failure (CHF) contributed to SRAEs. While sedation is advantageous in many cases, it also carries contraindications. Another study showed that SRAEs during endoscopic retrograde cholangiopancreatography (ERCP) in high-risk patients were more likely to occur under CS than GA. Specific SRAEs recorded included hypoxemia, need for airway maneuvers, hypotension requiring vasopressors, procedural interruptions related to inadequate sedation, cardiac arrhythmia, and respiratory failure [12]. One of the major risks with sedation is movement, so wrist restraints should be employed for TAVR.

In a trial of over 100 morbidly obese patients for ERCP or endoscopic ultrasound, a nasal trumpet was found to be as effective as BiPAP [13]. Continuous positive airway pressure (CPAP) and BiPAP can be added through a trumpet connected to the tip of an endotracheal tube and the regular anesthesia machine circuit using pressure support mode ventilation. Deep sedation in over 11,000 patients showed a similar risk of desaturation across all classes of obesity (I, II, and III), with an incidence of about 20% in obese patients compared to 13% in non-obese patients.

In another endoscopy study of hundreds of patients with an average BMI of 55.6 vs. 22.5, CS was satisfactory, with an average dose of fentanyl of 180 µg vs. 148 µg and midazolam of 7.7 mg vs. 6.4 mg. More hypoxemia in obese patients (5% vs. 0%) was likely related to more OSA (58% vs. 4%). Half of the obese patients required CPAP during endoscopy and also diphenhydramine rescue after inadequate sedation. Fortunately, one month later, no difference was noted in complications [14].

In this regard, another study found that 25% of patients with a BMI over 30 required conversion to GA due to respiratory complications [15]. Although advancements in anesthesia techniques have led to a decline in oversedation-related respiratory distress during moderate and deep sedation, insufficient monitoring can lead to oversedation. It is essential to closely monitor sedation levels and maintain vigilance throughout the procedure to prevent adverse events [16,17]. This suggests cost-effectiveness in requesting a cardiac anesthesiologist to be present solo in a TAVR with severe OSA.

Socioeconomic Status

When compared to wealthy white males, TAVR patients with other demographics have higher mortality [18]. Their mortality can be accompanied by comorbidities that predict the outcome. Common comorbidities include hypertension, CAD, diabetes mellitus, and chronic kidney disease. In one study, major adverse cardiac events (MACE) occurred in 21% of such critically ill patients during the follow-up period of around five years [19].

Patients undergoing coronary artery bypass graft surgery (CABG) have lower 30-day mortality rates when they receive long-term, well-coordinated care from primary care providers, undergo surgery at large-volume CABG centers, and reside in affluent counties [20]. Part of the cost advantage from large volumes within health maintenance organizations is attributable to preoperative optimization during monthly multidisciplinary, presurgical case-based meetings, which apply enhanced recovery after surgery pathways [20]. At such meetings, decisions can be triaged to CS, MAC, or GA. The strikingly low mortality rate at one such hospital, Kaiser Permanente, for CABG could be partially related to the one-on-one patient care by a cardiac anesthesiologist for each CABG. We submit that the explanation is that continuous presence enhances 360-degree closed communication. The anesthesia care team model is used at Kaiser in many other operating rooms, with one anesthesiologist to every four Certified Registered Nurse Anesthetists, particularly when there are fewer comorbidities and simpler procedures. Both patient- and surgery-related issues inevitably introduce errors in communication during the multiple handoffs inherent in supervising multiple anesthetizing locations.

A questionnaire in 2018 revealed that in 90% of TAVR cases, teams requested a perfusionist, surgeon, and cardiac anesthesiologist to be continuously present, with GA in 40%, MAC in 44%, and subcutaneous lidocaine with CS in 23%. Norepinephrine was the drug of choice, given in 63% of patients. TEE was used in fewer than 30% [21]. A cardiac anesthesiologist should be present throughout all high-risk TAVR procedures. Table 1 includes what the present authors deem high-risk [10,13,22-24]. Counties with poverty will not be able to recruit enough cardiac anesthesiologists (and other vital team members) to be present, so referral centers with large volumes tend to manifest better outcomes.

**Table 1:** Risk factors indicating the need for a cardiac anesthesiologist in TAVR. This table summarizes the various risk factors for transcatheter aortic valve replacement (TAVR).

Risk factors indicating the need for a cardiac anesthesiologist in TAVR
Severe pulmonary hypertension
Left ventricle ejection fraction less than 40%
STOP-Bang obstructive sleep apnea score over 3
Body mass index over 30
Dialysis
Home oxygen
Chronic or acute preoperative cardiovascular intravascular medications
Tolerance to opiates and benzodiazepines, such as those found in chronic pain states
Difficult TAVR cannulation sites (small calcified femoral artery)

In Germany, chest compressions for resuscitation, defibrillation, extra-corporeal membrane oxygenation, or conversion to GA were prospectively examined in a registry of over 600 TAVR. Nine percent had one of those critical events, and 5.65% converted to GA [22]. A similar scheduling dilemma exists in pediatric cardiac catheterization labs. In one retrospective study of children under two years of age, propensity scores were adjusted, finding 46% required GA and 54% required sedation [23]. Conversion rate to GA was 4%. Adverse events were more frequent in patients who were older or smaller, had undergone surgical palliation, had two ventricles rather than a single ventricle, were cyanotic, and - most importantly - had pulmonary hypertension (5.6-fold risk). Selecting CS did not affect the adverse event rate but demonstrated a lower need for vasopressors.

Congestive Heart Failure/Pulmonary Hypertension

Studies have shown that GA may be associated with higher rates of respiratory complications, particularly in patients with pre-existing cardiopulmonary diagnoses [23]. Pulmonary hypertension, because it causes the weaker, thinner right ventricle to more easily succumb to CHF, is the leading killer disease during conversion to GA, second only to left ventricular CHF. A North American registry described safety profiles of right heart catheterizations in children under MAC with a pediatric cardiac anesthesiologist. Risks were higher in older age, with higher carbon dioxide levels in blood gases, or requiring home oxygen or other medications for chronic pulmonary hypertension. Inhaled 20 ppm nitric oxide is often administered during catheterization as a challenge, alongside 100% inhaled oxygen, to determine whether pulmonary vascular resistance is reactive. When discontinuing the nitric oxide or oxygen too abruptly, rebound pulmonary hypertension crisis may occur, as can also occur during intubating or extubating too quickly. Cardiovascular collapse is best avoided by gently taking over positive pressure ventilation, while titrating vasopressors (epinephrine, dobutamine for adults, or dopamine for children) in a peripheral intravenous line. Many patients need an intensive care unit observation for 24 hours after just a simple cardiac catheterization, to monitor for pulmonary hypertension rebound, regardless of the type of anesthesia [24].

University of California, San Francisco studied over 450 pediatric right heart catheterizations to evaluate their severe pulmonary hypertension in response to medications between 2015 and 2020, with a goal of spontaneous breathing at a normal pH maintained by a pediatric cardiac anesthesiologist. Oral medications after such diagnostic catheterization can be useful if pulmonary hypertension proves reactive to nitric oxide and oxygen, and also if future surgeries are more safely permitted. More than 70% of these (average age of three years) did not need invasive airways, although 4% needed a rescue airway (with risks being age under one or vasoactive drugs at home). Under one-fifth of their patients received a planned invasive airway. The inhaled agent sevoflurane was utilized in half of all patients to establish an intravenous line. Dexmedetomidine infusion at approximately one µg/kg/h was the primary medication in 91% of children, midazolam supplements in 63%, ketamine in 63%, propofol in 38%, fentanyl in 28%, and sevoflurane in 64%. In the vast majority, just nasal prongs kept the airway open, but 6% required an LMA, and the remainder required an endotracheal tube. The one death reported was in a patient on home noninvasive CPAP and was planned to be intubated for the catheterization [25].

Research specifically after TAVR compared hundreds of patients on home oxygen therapy to thousands who were not. These patients often have concurrent pulmonary hypertension. The home oxygen group tended to be younger but had more pulmonary disease. Home oxygen patients often needed alternatives to femoral access for TAVR, had more cardiac arrest (4.7% vs. 1%), more atrial fibrillation (4% vs. 1.5%), more GA (51% vs. 36%), and more mortality at one year (17% vs. 7.5%) [26]. The choice during MAC of using propofol vs. dexmedetomidine has been extensively investigated. As expected, carbon dioxide increases by about 10 mmHg, but more especially at increased propofol doses or lower Bispectral Index (BIS). Younger patients, those with a higher BMI, and men also had higher carbon dioxide levels, which generally required the use of more vasopressors. From 2019 to 2022, no worse outcomes (in terms of length of stay and 30-day mortality) were noted with this mildly higher carbon dioxide level of about 55±10 mmHg standard deviation present during the valve deployment phase, when patient movement must be avoided. The BIS monitor's average at deployment was measured at 48. Pre-existing pulmonary hypertension was compared (at right ventricular systolic pressure groups of average 30 mmHg, 50 mmHg, and 70 mmHg), but higher amounts of pulmonary artery pressure were not associated with poor outcomes. However, low ejection fraction (CHF) and longer procedure duration were both associated with the use of more vasopressors. Smoking, obstructive lung disease, home oxygen therapy, and OSA were not associated with higher CO_2_ levels. The duration of the intensive care stay was related to vasopressor dose, renal insufficiency, and length of procedure. The biggest risk associated with length of hospitalization was age, duration of procedure, and renal insufficiency. Ironically, the same groups receiving the least anesthetic doses had longer stays. BIS and sedative agent selection did not affect the use of vasopressors. Propofol and remifentanil were linked to an incidence of 94% (vs. only 13% in GA) of pulmonary complications in a randomized controlled trial. Ketamine in this study was associated with higher carbon dioxide (although this could be because clinicians chose ketamine, knowing carbon dioxide was rising). This study lacked sufficient power due to its small sample size [27].

Cost-effectiveness analysis

Cost Analysis of Solo Anesthesiologist

The cost of one individual suffering permanent disability far exceeds the total savings gained by reducing costs for each patient across a few thousand patients. The cost of one cardiac anesthesiologist present for half a day of $1,000 is a small price to pay for safety. By default, when cardiologists request only CS, anesthesiologists are left out of that patient’s life. Comparing periods of 2008-2013, 2013-2017, and 2017-2022 in over 1,000 TAVR in one hospital, CS rose from zero to 60% and finally to 90%. The continuous presence of an anesthesiologist increased from 46% to 59% between the second and third periods. It is alarming that only having an anesthesiologist available to come in from far away (at outside buildings nearby) rose from 13.6% to 31.5%. Simultaneously, the use of GA was reduced from 38% to 8.3%. In over 90% of their TAVR procedures, access was through the femoral artery. The risk of urgent open sternotomy or aortic branch repair is approximately 1%. Studies in Israel and Germany found no adverse events at 30 days when anesthesiologists were only on-call. Dialysis preoperatively was present in 1.3% of the patients, and the mean BMI was 27, suggesting that the TAVR patients in this German study were relatively healthier than current United States groups [28].

Costs associated with a TAVR in each patient under GA are higher than those with CS [29]. Total direct costs for CS were only 73% of those for GA. A recent randomized controlled trial showed similar one-year outcomes regardless of CS or GA [30]. The lower demand for resources can also make it a more cost-effective option for hospitals [31]. Patients were followed for five years and randomized to receive either a Medtronic or Edwards valve, and then further randomized to CS or GA. The risk of mortality, stroke, myocardial infarction, or acute kidney injury at five years was 51% in CS and 61% in GA, but the p-value was only 0.09. All-cause mortality was 41% in CS vs. 54% in GA at five years. GA was usually remifentanil and propofol, and CS or MAC was usually dexmedetomidine or propofol. No difference in delirium was found, regardless of GA, compared to MAC or CS, reminiscent of a study that found no difference in functional neurologic status a year after neuroangiography interventions for thrombotic stroke [32].

Sedation is the preferred economic option for TAVR [33]. On the contrary, a few sources, for instance, a 2020 randomized trial (RCT) reported similar outcomes between the use of CS and GA, although the level of involvement of an anesthesiologist was not described in detail [34]. Baseline characteristics in a different RCT testing high-flow nasal oxygen had a mean age of 82 but a BMI of only 27 [35]. It is unclear whether a review showing a risk ratio of 0.37 for reducing hypoxemia and 0.26 for minor airway assistance matters in obese patients, as they found no impact on hypercarbia [36]. A different randomized controlled trial (RCT) was halted after it showed that high-flow nasal cannula at 60 L/min with an FiO₂ of 36-40% did not result in overall clinical improvement compared to standard nasal prongs at 4 L/min [37].

Tied to preventing OSA is the trend, whether CS or MAC, of relying upon dexmedetomidine. An intriguing study paired speaking comforting comments and playing relaxing music while others received remifentanil infusion. The incidence of postoperative delirium was 12%, but it was lower when music was played or when good comments were exchanged. More delirium was associated with dialysis, pulmonary hypertension, pulmonary disease, diabetes, and moderate dementia. Average BIS was 44 [38]. Dexmedetomidine was usually at 0.5 µg/kg/h in a study with either BIS target 40 if deep or 55 for light assigned groups. Dexmedetomidine seems to reduce the risk of delirium in half [39]. In many patients, CS is not an option. For example, in one study of over 400 patients, CS was unlikely to be chosen if the CHF New York Heart Association Class was over 2, valve-in-valve TAVR, or a self-expanding valve [40].

In that study with more critically ill patients in the GA group, it is not surprising that fewer blood transfusions, procedure time, and inotropes were used in the CS group. However, at one month, no difference was found in a composite rate of 18% for death, stroke, transient ischemic attack, myocardial infarction, newly required dialysis, major vascular complication, pacemaker placement, or bleeding [40]. Bias in that study was introduced by retrospective data and a trend to choose less GA over the years, as well as the fact that critically ill patients often require GA [40]. Minimizing staffing to exclude an anesthesiologist during CS means clinicians must triage those with OSA, pulmonary hypertension, and CHF to MAC ([41]. Other researchers have found that with CS, 30-day readmission rates are not different, but costs are lower. Same-day discharge is more common for CS [42]. Choice of anesthesia technique is associated with earlier hospital discharge and reduced costs after TAVR [43]. They found that from 2012 to 2017, the cost was almost $56,000 for GA or $50,000 for CS. No difference in preoperative comorbidities, including pulmonary disease or CHF, was noted between CS and GA. Contraindications for minimalist CS often include respiratory distress, musculoskeletal pain, agitation, or more difficult vascular approaches [44].

In a high-volume single-center study, a 2020 review found that only 29% of TAVR cases had an anesthesiologist on site, with the majority having only one anesthesiologist on call. Only under 2% of patients required an anesthesiologist to rescue cardiorespiratory issues urgently [45]. Questionnaires show that dexmedetomidine is most common in about 85% of all patients and only achieves light CS levels. The conversion to GA rate was 10%, with only one reason being hypercarbia [46]. Anesthesiologists often use more dexmedetomidine than midazolam, as it has been shown to improve the airway after general surgeries as well [47]. However, dexmedetomidine is not a panacea. In OSA patients in a prospective trial, dexmedetomidine caused more pharyngeal collapse than natural sleep [48]. OSA is one of the highest risk comorbidities, linked to a 1.7-fold greater odds of adverse events, 3.3-fold odds of requiring airway intervention during other procedures [49].

Cost of Urgent Conversion to General Anesthesia

Conversion to emergent GA must be avoided to prevent permanent disability. A corollary can be seen in children with severe heart disease, where the overall incidence of cardiac arrest after non-cardiac surgery was 0.1%, with an intraoperative rate of 0.05% and a 48-h postoperative rate of 0.06%. Significant risk factors for perioperative cardiac arrest include infancy, American Society of Anesthesiology Physical Status classification, admission through the emergency room, inpatients, major cardiac disease, impaired cognitive status, and longer anesthesia duration. Perioperative cardiac arrest was significantly associated with longer hospital length of stay, reoperation, differences in discharge destination, and 30-day mortality. In addition, patients experiencing postoperative cardiac arrest had a significantly higher rate of in-hospital and 30-day mortality.

Lower 30-day mortality after intraoperative cardiac arrest can occur after prompt recognition and rapid initiation of intraoperative resuscitation. Identification of perioperative risk factors for cardiac arrest is crucial to safety. A 2019 database reported perioperative cardiac arrest in children and found 34% had congenital heart disease, such as a single ventricle, especially pre-Fontan, severe pulmonary hypertension (3x risk, so postoperatively likely need critical care unit), complex (for example, severe aortic stenosis, or cyanotic), or a low ejection fraction. The chances of events were about 30% higher in the recovery phase than intraoperatively, especially when GA was used or surgery was an emergency [50].

In over 500 surgeries on adults who already had a Fontan procedure, the MAC was 48%, the GA was 52%, and the CS was 20%. Ninety-three complications occurred and included a few of each: arrhythmia requiring intervention, hypotension, bradycardia, hypoxia, heart failure requiring intravenous diuretics, acute kidney injury, bleeding requiring blood transfusion, unplanned procedures for dialysis catheter placement, readmission, unplanned hospitalization for hypoxia, and unplanned transfer to the intensive care unit. Baseline cyanosis was the only risk factor for complications. Procedural complications were more common in the Fontan group (at 18%) than in the 2-ventricle congenital heart disease group (at 5%) and the no heart disease group (at 1.4%). All-cause mortality was low and may be related to the multidisciplinary monthly meetings. This approach improves last-minute surgery cancellations for further work-ups and the timely declaration of the type of anesthesia. Mayo Clinic reported other procedures in hundreds of patients after Fontan operations, and revealed CHF or hypoxemia were the outstanding risk factors; these two groups should likewise be triaged earlier to a solo cardiac anesthesiologist, to prevent urgent conversion to GA [51].

One paper sought to develop a simple score, using preprocedural variables, for the prediction of 30-day mortality after TAVR. Patients from a national multicenter registry who underwent TAVR were randomly assigned by baseline characteristics [52]. A similarly constructed study of perioperative risk in congenital heart disease, in which 78% required GA, described that the greatest preoperative comorbidity was supra-systemic pulmonary artery pressure. Cardiac arrest was almost twice as common postoperatively, especially with prolonged QT interval, CAD, or severe AS. Only moderate levels of risk were present in cases of difficult airway or OSA, moderate AS, moderate pulmonary stenosis, or other factors [53]. A prospective study defined moderate risk as Wolff-Parkinson-White syndrome, a pacemaker, a chronically prolonged QT interval, simple unrepaired cardiac disease, or repaired complex cardiac disease. In contrast, high-risk factors included unrepaired complex disease, severe valve disease, severe pulmonary hypertension, mechanical ventricular assist devices, severe low left ventricular ejection fraction, preoperative ventilator use, kidney injury, vasopressor use, emergency surgery, and advanced age [53].

In summary, be aware that by 2017, CS had increased from 0% to 63%. Higher Society of Thoracic Surgeons illness scores in those with TEE (6.34% vs 4.45%) remind one that the sicker patients may still not be qualified for just CS. Patients with increased age and CAD had an increased risk for mortality, whereas those who had undergone coronary artery bypass grafting (CABG) had a decreased mortality [54].

Similar to the authors in the Journal of Clinical Anesthesia, when children with congenital heart disease require general anesthesia for general surgeries, we aimed in this cost-benefit analysis to provide a risk scoring system [53]. Saettele et al. found that a pediatric cardiac fellowship-trained anesthesiology physician should take care of all children in high-risk general surgeries, including those with single ventricle physiology, severe pulmonary hypertension, and severe aortic stenosis [53]. The goal of this review was to determine the ease of use of a preoperative risk stratification score. This score could save financial and personnel costs because a higher score suggests assigning a cardiac anesthesia fellowship-trained physician to work alone to provide anesthesia. If the risk score is low, this indicates that a conscious sedation nurse would provide adequate safety at a lower cost. This score will categorize and triage which high-risk patients would benefit from the more costly staffing of a physician alone. In this era of cost containment, it is essential that an anesthesiology department have one physician who schedules the staff for the following day’s surgeries. This physician should consider reviewing the chart to determine the risk of major adverse postoperative complications and assign the corresponding staffing levels according to Table 2. General anesthesia in the hands of a fellowship-trained physician can be safer than sedation, as found in the six-month follow-up (excellent morbidity, mortality, and neurologic function) after massive ischemic stroke neurointerventional cases [32]. This was believed to be due to holding still during the procedure and to the physician maintaining excellent cardiac output and blood pressure for the perfusion of all organs (Table 2).

**Table 2:** Suggested novel point-based scoring system to assess total risk. OSA: obstructive sleep apnea; CPAP: continuous positive airway pressure; BMI: body mass index; PulmHTN: pulmonary artery hypertension; LV EF: left ventricular ejection fraction; AS: aortic stenosis; PAD: peripheral arterial disease; RN: registered nurse; CRNA: certified registered nurse anesthetist; sev: severe PulmHTN moderate is defined as pulmonary artery systolic pressure >50% of systemic systolic blood pressure, and severe PulmHTN is defined as pulmonary artery systolic pressure >65% of systemic systolic blood pressure. Patients receiving hemodialysis for end-stage renal failure or peritoneal dialysis are included. AS is considered significant when the mean gradient is >45 mmHg, the peak gradient is >70 mmHg, or the valve area is <0.6 cm². Severe PAD is noted in the chart when femoral artery access is difficult, such as in patients with prior procedures to open peripheral arteries. A CRNA is defined as a provider who has completed two years of intensive care RN experience and three years of graduate school. Severe carotid stenosis is defined as >70% narrowing of one carotid artery.

Risk factors	Points
OSA on CPAP	3
OSA no CPAP	2
BMI over 40	2
BMI 30-40	1
Mod-PulmHTN	4
Sev-PulmHTN	7
Dilated RV moderate-sev	4
LV EF <25%	3
Hemodialysis	2
Age >80 years	2
Severe AS	2
Severe PAD access for TAVR placement artery difficult	4
Severe carotid stenosis	3

Complex cardiac surgery at Kaiser, a Health Maintenance Organization (HMO), is provided as one-on-one (reducing information lost in handoffs) patient care by fellowship-trained cardiac anesthesia physicians, with great outcomes. At these same HMOs, less complex vascular surgery for patients with fewer comorbidities is performed by staff with fewer years of training, but supervised by an anesthesia physician, resulting in great outcomes. In contrast, many hospitals have one general anesthesia physician supervising four cardiac surgeries and four nurse anesthetists, which may lead to worse outcomes. An HMO is better able to set standards after analyzing its outcome data and appointing an anesthesia director to create policy for a much larger volume center [20].

## Conclusions

Three main points highlight the implications of triage to minimalist CS compared to MAC or GA with an anesthesiologist continuously present during TAVR. Firstly, CHF, especially pulmonary hypertension, requires a cardiac anesthesiologist to titrate vasopressors as well as different sedatives. Secondly, obstructive sleep apnea requires MAC. Finally, additional monitors can prevent complications when paired with titration of medications, such as BIS, cerebral oximetry, and BiPAP devices. While CS is adequate for most patients, those with CHF and OSA require an anesthesiologist. It is imperative to discuss each patient at a monthly multidisciplinary meeting.
